# 3-Hydr­oxy-7,8-dimethoxy­quinolin-2(1*H*)-one

**DOI:** 10.1107/S1600536808011549

**Published:** 2008-04-30

**Authors:** Jian Song, Yongcheng Lin, Wing Lai Chan

**Affiliations:** a School of Pharmacy, Guangdong Pharmaceutical University, Guangzhou 510006, People’s Republic of China; b School of Chemistry and Chemical Engineering, Sun Yat-sun University, Guangzhou 510275, People’s Republic of China; c Department of Applied Biology and Chemistry Technology, Polytechnic University of Hong Kong, Hong Kong, People’s Republic of China

## Abstract

In the crystal structure of the title compound, C_11_H_11_NO_4_, intra­molecular O—H⋯O hydrogen bonding results in the formation of a planar five-membered ring, which is nearly coplanar with the quinoline group. Inter­molecular N—H⋯O hydrogen bonds link the mol­ecules into centrosymmetric dimers.

## Related literature

For general background, see: Beak (1977 ▶); Nimlos *et al.* (1987 ▶); Rajnikant *et al.* (2002 ▶); Johnson (1996 ▶). For related literature, see: Lin *et al.* (2000 ▶); Song *et al.* (2006 ▶).
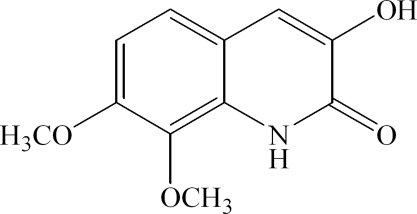


## Experimental

### 

#### Crystal data

C_11_H_11_NO_4_
*M* *_r_* = 221.21
Monoclinic, 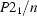

*a* = 4.9655 (16) Å
*b* = 14.084 (5) Å
*c* = 14.888 (5) Å
β = 96.208 (6)°
*V* = 1035.1 (6) Å^3^
*Z* = 4
Mo *K*α radiation
μ = 0.11 mm^−1^
*T* = 294 (2) K
0.60 × 0.37 × 0.31 mm

#### Data collection

Bruker SMART CCD area-detector diffractometer
Absorption correction: multi-scan (*SADABS*; Sheldrick, 1996 ▶) *T*_min_ = 0.937, *T*_max_ = 0.967
6788 measured reflections
2228 independent reflections
1761 reflections with *I* > 2σ(*I*)
*R*_int_ = 0.015

#### Refinement

*R*[*F* ^2^ > 2σ(*F* ^2^)] = 0.053
*wR*(*F* ^2^) = 0.174
*S* = 1.08
2228 reflections
150 parameters
H atoms treated by a mixture of independent and constrained refinement
Δρ_max_ = 0.50 e Å^−3^
Δρ_min_ = −0.25 e Å^−3^

### 

Data collection: *SMART* (Bruker, 1998 ▶); cell refinement: *SMART*; data reduction: *SAINT* (Bruker, 1999 ▶); program(s) used to solve structure: *SHELXS97* (Sheldrick, 2008 ▶); program(s) used to refine structure: *SHELXL97* (Sheldrick, 2008 ▶); molecular graphics: *SHELXTL* (Sheldrick, 2008 ▶); software used to prepare material for publication: *SHELXTL*.

## Supplementary Material

Crystal structure: contains datablocks global, I. DOI: 10.1107/S1600536808011549/hk2455sup1.cif

Structure factors: contains datablocks I. DOI: 10.1107/S1600536808011549/hk2455Isup2.hkl

Additional supplementary materials:  crystallographic information; 3D view; checkCIF report

## Figures and Tables

**Table 1:** Hydrogen-bond geometry (Å, °)

*D*—H⋯*A*	*D*—H	H⋯*A*	*D*⋯*A*	*D*—H⋯*A*
N1—H1⋯O1^i^	0.90 (3)	2.07 (3)	2.938 (2)	161 (2)
O2—H2⋯O1	0.82	2.33	2.756 (2)	113

Symmetry code: (i) 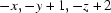.

**Fig. 1.**
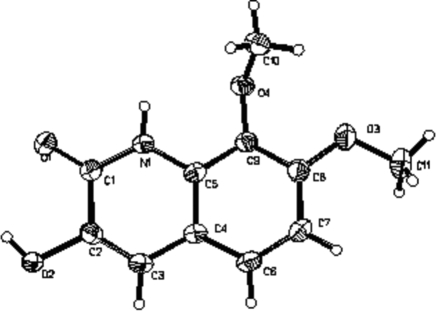
The molecular structure of the title molecule, with the atom-numbering scheme. Displacement ellipsoids are drawn at the 50% probability level.

**Fig. 2.**
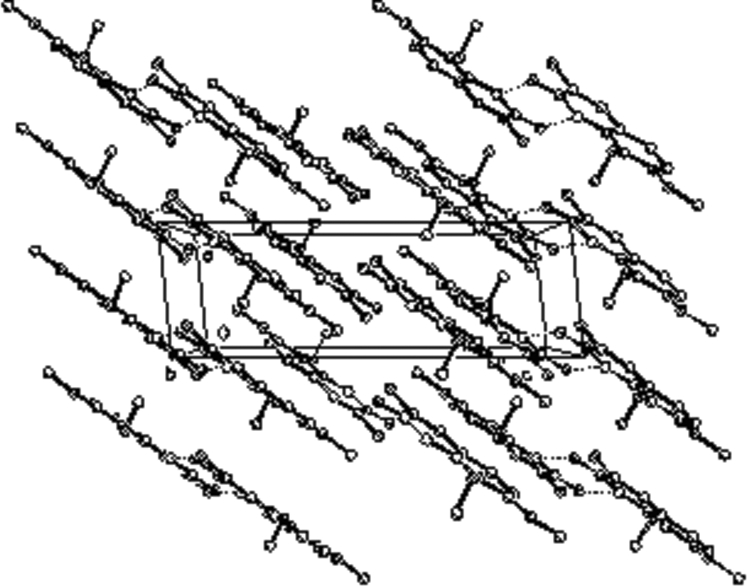
A partial packing diagram of (I). Hydrogen bonds are shown as dashed lines.

## supplementary crystallographic information

### Comment

Quinolin-2(1*H*)-ones can exist in both the lactam and lactim forms (Beak, 1977; Nimlos *et al.*,  1987; Rajnikant *et al.*, 2002). The tautomeric equilibrium of lactam-lactim attracts attention owing to its chemical, biological and theoretical importantce (Johnson, 1996). The title compound, (I), which is a part of the marine natural compound penicilliazine (Lin *et al.*, 2000), was synthesized and characterized by our research group toward the natural product total synthesis. As part of our ongoing studies, we report herein the crystal structure of (I).

The molecule of the title compound, (I), (Fig. 1) adopts a bicyclic lactam-form with one hydroxy and two methoxy groups attached to atoms C2, C8 and C9, respectively. Rings A (N1/C1-C5) and B (C4-C9) are, of course, planar and the dihedral angle between them is A/B = 2.18 (3)°. The intramolecular O-H···O hydrogen bond (Table 1) results in the formation of a planar five-membered ring C (O1/O2/H2/C1/C2). Ring C is oriented with respect to the adjacent rings A and B at dihedral angles of A/C = 1.99 (3)° and B/C = 3.96 (3)°. So, rings A, B and C are nearly coplanar.

In the crystal structure, intermolecular N-H···O hydrogen bonds (Table 1) link the molecules into centrosymmetric dimers (Fig. 2), in which they may be effective in the stabilization of the structure.

### Experimental

The title compound, (I), was prepared according to our reported procedure (Song *et al.*,  2006). Suitable crystals were obtained by recrystallization from chloroform/ethyl acetate (1:1) solution (m.p. 436-437 K). Spectroscopic analysis: IR (KBr, νcm^-1^): 3442, 3169, 1665, 1638, 1116; ^1^H NMR (CDCl_3_, δ, p.p.m.): 7.14–7.17(d, 1H, J = 9.0 Hz), 7.07(s, 1H), 6.85-6.88 (d, 1H, J = 9.0 Hz), 6.61(br, OH),3.97 (s, 3H), 3.93 (s, 3H); ^13^C NMR (CDCl_3_, δ, p.p.m.): 159.0, 150.2, 143.7, 134.2,127.2,121.1,115.7,112.2, 108.8,60.6,56.0; analysis, calculated for C_11_H_11_N_1_O_4_: C 59.73, H 5.01, N 6.33%; found: C 59.98, H 5.23, N 6.14%.

### Refinement

H atom (for NH) was located in a difference syntheses and refined [N-H = 0.90 (3) Å and U_iso_(H) = 0.068 (7) Å^2^]. The remaining H atoms were positioned geometrically, with O-H = 0.82 Å (for OH) and C-H = 0.93 and 0.96 Å for aromatic and methyl H, respectively, and constrained to ride on their parent atoms with U_iso_(H) = xU_eq_(C,O), where x = 1.2 for aromatic H, and x = 1.5 for all other H atoms.

### Crystal data

**Table:**

C_11_H_11_NO_4_	*F*_000_ = 464
*M**_r_* = 221.21	*D*_x_ = 1.420 Mg m^−^^3^
Monoclinic, *P*2_1_/*n*	Mo *K*α radiation λ = 0.71073 Å
Hall symbol: -P 2yn	Cell parameters from 886 reflections
*a* = 4.9655 (16) Å	θ = 3.1–27.1º
*b* = 14.084 (5) Å	µ = 0.11 mm^−^^1^
*c* = 14.888 (5) Å	*T* = 294 (2) K
β = 96.208 (6)º	Block, colorless
*V* = 1035.1 (6) Å^3^	0.60 × 0.37 × 0.31 mm
*Z* = 4	

### Data collection

**Table:**

Bruker CCD area-detector diffractometer	2228 independent reflections
Radiation source: fine-focus sealed tube	1761 reflections with *I* > 2σ(*I*)
Monochromator: graphite	*R*_int_ = 0.015
*T* = 294(2) K	θ_max_ = 27.1º
φ and ω scans	θ_min_ = 2.0º
Absorption correction: multi-scan(SADABS; Sheldrick, 1996)	*h* = −6→5
*T*_min_ = 0.937, *T*_max_ = 0.967	*k* = −17→15
6788 measured reflections	*l* = −18→18

### Refinement

**Table:**

Refinement on *F*^2^	Secondary atom site location: difference Fourier map
Least-squares matrix: full	Hydrogen site location: inferred from neighbouring sites
*R*[*F*^2^ > 2σ(*F*^2^)] = 0.054	H atoms treated by a mixture of independent and constrained refinement
*wR*(*F*^2^) = 0.174	*w* = 1/[σ^2^(*F*_o_^2^) + (0.0907*P*)^2^ + 0.3738*P*] where *P* = (*F*_o_^2^ + 2*F*_c_^2^)/3
*S* = 1.08	(Δ/σ)_max_ < 0.001
2228 reflections	Δρ_max_ = 0.50 e Å^−^^3^
150 parameters	Δρ_min_ = −0.25 e Å^−^^3^
Primary atom site location: structure-invariant direct methods	Extinction correction: none

### Special details

**Table:**

Geometry. All e.s.d.'s (except the e.s.d. in the dihedral angle between two l.s. planes) are estimated using the full covariance matrix. The cell e.s.d.'s are taken into account individually in the estimation of e.s.d.'s in distances, angles and torsion angles; correlations between e.s.d.'s in cell parameters are only used when they are defined by crystal symmetry. An approximate (isotropic) treatment of cell e.s.d.'s is used for estimating e.s.d.'s involving l.s. planes.
Refinement. Refinement of F^2^ against ALL reflections. The weighted R-factor wR and goodness of fit S are based on F^2^, conventional R-factors R are based on F, with F set to zero for negative F^2^. The threshold expression of F^2^ > 2sigma(F^2^) is used only for calculating R-factors(gt) etc. and is not relevant to the choice of reflections for refinement. R-factors based on F^2^ are statistically about twice as large as those based on F, and R- factors based on ALL data will be even larger.

### Fractional atomic coordinates and isotropic or equivalent isotropic displacement parameters (Å^2^)

**Table:**

	*x*	*y*	*z*	*U*_iso_*/*U*_eq_	
O1	−0.1615 (3)	0.39255 (11)	1.01266 (11)	0.0616 (5)	
O2	−0.2273 (3)	0.20136 (9)	0.97741 (9)	0.0514 (4)	
H2	−0.2816	0.2327	1.0182	0.077*	
O3	0.6647 (4)	0.48962 (12)	0.67193 (12)	0.0681 (5)	
O4	0.3678 (3)	0.54717 (9)	0.80367 (10)	0.0498 (4)	
N1	0.1136 (3)	0.41905 (11)	0.90243 (11)	0.0439 (4)	
H1	0.119 (5)	0.481 (2)	0.9155 (17)	0.068 (7)*	
C1	−0.0380 (4)	0.36225 (14)	0.95063 (13)	0.0458 (5)	
C2	−0.0452 (4)	0.26212 (14)	0.92587 (13)	0.0483 (5)	
C3	0.0957 (4)	0.22783 (14)	0.86147 (14)	0.0500 (5)	
H3A	0.0912	0.1632	0.8488	0.060*	
C4	0.2532 (4)	0.28997 (13)	0.81204 (13)	0.0442 (4)	
C5	0.2543 (4)	0.38754 (13)	0.83327 (12)	0.0404 (4)	
C6	0.4035 (5)	0.26022 (15)	0.74343 (15)	0.0535 (5)	
H6A	0.4079	0.1960	0.7290	0.064*	
C7	0.5456 (5)	0.32376 (16)	0.69650 (15)	0.0544 (5)	
H7A	0.6462	0.3021	0.6514	0.065*	
C8	0.5392 (4)	0.42049 (15)	0.71632 (14)	0.0490 (5)	
C9	0.3942 (4)	0.45222 (12)	0.78474 (13)	0.0427 (4)	
C10	0.6032 (5)	0.58783 (16)	0.85291 (18)	0.0617 (6)	
H10A	0.5711	0.6538	0.8638	0.093*	
H10B	0.7547	0.5816	0.8184	0.093*	
H10C	0.6416	0.5554	0.9095	0.093*	
C11	0.8222 (6)	0.4614 (2)	0.60194 (19)	0.0789 (8)	
H11A	0.8986	0.5166	0.5766	0.118*	
H11B	0.7088	0.4286	0.5557	0.118*	
H11C	0.9653	0.4200	0.6265	0.118*	

### Atomic displacement parameters (Å^2^)

**Table:**

	*U*^11^	*U*^22^	*U*^33^	*U*^12^	*U*^13^	*U*^23^
O1	0.0712 (10)	0.0546 (9)	0.0639 (9)	−0.0102 (7)	0.0302 (8)	−0.0133 (7)
O2	0.0809 (10)	0.0349 (7)	0.0397 (7)	−0.0015 (6)	0.0119 (6)	−0.0009 (5)
O3	0.0819 (12)	0.0579 (10)	0.0713 (10)	0.0009 (8)	0.0395 (9)	0.0048 (7)
O4	0.0549 (8)	0.0341 (7)	0.0615 (8)	0.0020 (6)	0.0117 (6)	−0.0013 (6)
N1	0.0498 (9)	0.0337 (8)	0.0496 (9)	−0.0009 (6)	0.0118 (7)	−0.0058 (6)
C1	0.0491 (10)	0.0431 (10)	0.0463 (10)	−0.0035 (8)	0.0096 (8)	−0.0056 (8)
C2	0.0559 (12)	0.0403 (10)	0.0488 (10)	−0.0069 (8)	0.0063 (9)	0.0001 (8)
C3	0.0620 (12)	0.0332 (9)	0.0552 (11)	−0.0018 (8)	0.0076 (9)	−0.0039 (8)
C4	0.0489 (10)	0.0360 (9)	0.0480 (10)	0.0022 (8)	0.0058 (8)	−0.0042 (7)
C5	0.0415 (9)	0.0371 (9)	0.0427 (9)	0.0037 (7)	0.0045 (7)	−0.0038 (7)
C6	0.0606 (13)	0.0398 (10)	0.0611 (12)	0.0061 (9)	0.0115 (10)	−0.0110 (9)
C7	0.0580 (12)	0.0521 (12)	0.0557 (11)	0.0083 (9)	0.0177 (9)	−0.0081 (9)
C8	0.0507 (11)	0.0471 (11)	0.0507 (11)	0.0033 (8)	0.0129 (9)	0.0029 (8)
C9	0.0452 (10)	0.0353 (9)	0.0480 (10)	0.0039 (7)	0.0061 (8)	−0.0005 (7)
C10	0.0622 (14)	0.0469 (11)	0.0779 (15)	−0.0108 (10)	0.0157 (11)	−0.0078 (10)
C11	0.0848 (18)	0.0863 (19)	0.0728 (16)	−0.0045 (15)	0.0419 (14)	0.0001 (14)

### Geometric parameters (Å, °)

**Table:**

O2—H2	0.8200	C5—C4	1.410 (3)
O3—C11	1.425 (3)	C6—C7	1.376 (3)
O4—C9	1.376 (2)	C6—C4	1.393 (3)
O4—C10	1.430 (3)	C6—H6A	0.9300
N1—C1	1.356 (3)	C7—H7A	0.9300
N1—C5	1.379 (2)	C8—O3	1.365 (3)
N1—H1	0.90 (3)	C8—C9	1.384 (3)
C1—O1	1.238 (2)	C8—C7	1.395 (3)
C1—C2	1.457 (3)	C10—H10A	0.9600
C2—O2	1.513 (2)	C10—H10B	0.9600
C3—C2	1.337 (3)	C10—H10C	0.9600
C3—C4	1.430 (3)	C11—H11A	0.9600
C3—H3A	0.9300	C11—H11B	0.9600
C5—C9	1.394 (3)	C11—H11C	0.9600
			
C2—O2—H2	109.5	C4—C6—H6A	119.3
C8—O3—C11	118.1 (2)	C6—C7—C8	120.21 (19)
C9—O4—C10	113.78 (16)	C6—C7—H7A	119.9
C1—N1—C5	124.09 (16)	C8—C7—H7A	119.9
C1—N1—H1	117.9 (17)	O3—C8—C9	115.29 (18)
C5—N1—H1	118.0 (17)	O3—C8—C7	124.92 (19)
O1—C1—N1	122.64 (18)	C9—C8—C7	119.78 (19)
O1—C1—C2	121.43 (18)	O4—C9—C8	122.25 (17)
N1—C1—C2	115.93 (17)	O4—C9—C5	117.72 (17)
C3—C2—C1	122.03 (18)	C8—C9—C5	119.91 (17)
C3—C2—O2	123.18 (17)	O4—C10—H10A	109.5
C1—C2—O2	114.78 (17)	O4—C10—H10B	109.5
C2—C3—C4	120.40 (18)	H10A—C10—H10B	109.5
C2—C3—H3A	119.8	O4—C10—H10C	109.5
C4—C3—H3A	119.8	H10A—C10—H10C	109.5
C6—C4—C5	117.91 (18)	H10B—C10—H10C	109.5
C6—C4—C3	124.03 (18)	O3—C11—H11A	109.5
C5—C4—C3	118.06 (17)	O3—C11—H11B	109.5
N1—C5—C9	119.87 (16)	H11A—C11—H11B	109.5
N1—C5—C4	119.41 (17)	O3—C11—H11C	109.5
C9—C5—C4	120.72 (17)	H11A—C11—H11C	109.5
C7—C6—C4	121.43 (18)	H11B—C11—H11C	109.5
C7—C6—H6A	119.3		
			
C10—O4—C9—C8	−77.3 (2)	C9—C5—C4—C3	−177.13 (18)
C10—O4—C9—C5	106.8 (2)	N1—C5—C9—O4	−5.1 (3)
C5—N1—C1—O1	179.73 (19)	C4—C5—C9—O4	174.46 (17)
C5—N1—C1—C2	0.4 (3)	N1—C5—C9—C8	178.93 (17)
C1—N1—C5—C9	176.93 (18)	C4—C5—C9—C8	−1.6 (3)
C1—N1—C5—C4	−2.6 (3)	C7—C6—C4—C5	−1.1 (3)
O1—C1—C2—C3	−177.3 (2)	C7—C6—C4—C3	178.2 (2)
N1—C1—C2—C3	2.0 (3)	C4—C6—C7—C8	−0.7 (3)
O1—C1—C2—O2	3.7 (3)	C9—C8—O3—C11	178.7 (2)
N1—C1—C2—O2	−176.97 (16)	C7—C8—O3—C11	−2.2 (4)
C4—C3—C2—C1	−2.1 (3)	O3—C8—C7—C6	−177.6 (2)
C4—C3—C2—O2	176.75 (17)	C9—C8—C7—C6	1.4 (3)
C2—C3—C4—C6	−179.4 (2)	O3—C8—C9—O4	3.0 (3)
C2—C3—C4—C5	−0.1 (3)	C7—C8—C9—O4	−176.09 (19)
N1—C5—C4—C6	−178.29 (18)	O3—C8—C9—C5	178.85 (17)
C9—C5—C4—C6	2.2 (3)	C7—C8—C9—C5	−0.3 (3)
N1—C5—C4—C3	2.4 (3)		

### Hydrogen-bond geometry (Å, °)

**Table:**

*D*—H···*A*	*D*—H	H···*A*	*D*···*A*	*D*—H···*A*
N1—H1···O1^i^	0.90 (3)	2.07 (3)	2.938 (2)	161 (2)
O2—H2···O1	0.82	2.33	2.756 (2)	113

Symmetry codes: (i) −*x*, −*y*+1, −*z*+2.
